# Virus Evolution Reveals an Exclusive Role for LEDGF/p75 in Chromosomal Tethering of HIV

**DOI:** 10.1371/journal.ppat.0030047

**Published:** 2007-03-30

**Authors:** Anneleen Hombrouck, Jan De Rijck, Jelle Hendrix, Linos Vandekerckhove, Arnout Voet, Marc De Maeyer, Myriam Witvrouw, Yves Engelborghs, Frauke Christ, Rik Gijsbers, Zeger Debyser

**Affiliations:** 1 The Laboratory for Molecular Virology and Gene Therapy, KULeuven and IRC KULAK, Leuven, Flanders, Belgium; 2 The Laboratory for Biomolecular Dynamics, KULeuven, Leuven, Flanders, Belgium; 3 The Laboratory for Biomolecular Modelling, KULeuven, Leuven, Flanders, Belgium; King's College London, United Kingdom

## Abstract

Retroviruses by definition insert their viral genome into the host cell chromosome. Although the key player of retroviral integration is viral integrase, a role for cellular cofactors has been proposed. Lentiviral integrases use the cellular protein LEDGF/p75 to tether the preintegration complex to the chromosome, although the existence of alternative host proteins substituting for the function of LEDGF/p75 in integration has been proposed. Truncation mutants of LEDGF/p75 lacking the chromosome attachment site strongly inhibit HIV replication by competition for the interaction with integrase. In an attempt to select HIV strains that can overcome the inhibition, we now have used T-cell lines that stably express a C-terminal fragment of LEDGF/p75. Despite resistance development, the affinity of integrase for LEDGF/p75 is reduced and replication kinetics in human primary T cells is impaired. Detection of the integrase mutations A128T and E170G at key positions in the LEDGF/p75–integrase interface provides in vivo evidence for previously reported crystallographic data. Moreover, the complementary inhibition by LEDGF/p75 knockdown and mutagenesis at the integrase–LEDGF/p75 interface points to the incapability of HIV to circumvent LEDGF/p75 function during proviral integration. Altogether, the data provide a striking example of the power of viral molecular evolution. The results underline the importance of the LEDGF/p75 HIV-1 interplay as target for innovative antiviral therapy. Moreover, the role of LEDGF/p75 in targeting integration will stimulate research on strategies to direct gene therapy vectors into safe landing sites.

## Author Summary

Viruses have a limited genome and therefore exploit the cellular machinery of infected host cells to complete the replication cycle. Today there is a growing interest to unravel these virus–host interactions. Lentiviral integrases use the cellular protein LEDGF/p75 to tether the preintegration complex to the chromosome, although the existence of alternative host proteins substituting for the function of LEDGF/p75 has been proposed. Here, we used virus evolution to investigate the role and importance of LEDGF/p75 in HIV integration and replication. We selected a virus that is resistant against overexpression of the integrase binding domain of LEDGF/p75. This virus displays a reduced affinity for its own cofactor but still depends on the cofactor for replication. We demonstrate the unique and essential role of LEDGF/p75 in HIV replication and support the LEDGF/p75 HIV-1 interplay as target for innovative antiviral therapy. Moreover, elucidation of the role of LEDGF/p75 in targeting integration will stimulate research on strategies to direct gene therapy vectors into safe landing sites.

## Introduction

Although a dynamic interplay between the virus and host factors occurs at all steps of the HIV replication cycle, the identity and role of these cellular cofactors are poorly understood. LEDGF/p75 is a cellular cofactor of HIV-1 integrase (IN), which catalyses integration of the HIV-1 DNA into the host cell genome [1]. LEDGF/p75 was found to interact with HIV-1 IN following coimmunoprecipitation of nuclear extracts of cells stably expressing IN from a synthetic gene [2]. The tight binding between LEDGF/p75 and IN was corroborated independently [3,4] and found to be lentivirus specific [5–7]. The protein of 530 amino acids contains a PWWP domain at the N terminus that may be involved in chromatin binding [8,9]. The interaction of LEDGF/p75 with IN is mediated by a minimal IN-binding domain (IBD) [10,11], in the C-terminal region of the protein (amino acids 347 to 429). LEDGF/p75 appears to function as a tethering factor, linking IN to the chromatin [12,13]. A recent report suggested a modest contribution of LEDGF/p75 to the targeting of HIV-1 integration, implying a role for alternative cofactors [14].

Several studies have addressed the question whether LEDGF/p75 is an important cofactor for viral replication in vivo. The Q168 residue in HIV-1 IN was found to be critical for the interaction with LEDGF/p75 [4]. A virus clone containing this single point mutation was defective for replication due to a block at the integration step [4]. Two studies failed to observe a reduction in HIV-1 replication after partial knockdown of LEDGF/p75 [6,15]. Vandekerckhove et al. [16], however, proved that more potent, transient or stable knockdown of LEDGF/p75 results in a significant inhibition of HIV-1 replication in different cell lines. These data have been independently confirmed [17,18]. The most potent inhibition of HIV-1 replication was observed upon stable overexpression of C-terminal fragments of LEDGF/p75 fused to the enhanced green fluorescent protein (eGFP). These fragments compete with LEDGF/p75 for binding to IN. The absence of chromatin interaction domains renders these fragments dysfunctional for tethering integration [19].

In this study, we show that HIV can develop resistance to overexpressed truncation variants of its own cellular cofactor, although at the expense of a reduced affinity to LEDGF/p75 and highly reduced replication kinetics. To our knowledge, such resistance phenotype to a cellular cofactor has not been described before. Detection of mutations at key positions in the LEDGF/p75–IN interface provides critical evidence of the importance of LEDGF/p75 for viral replication.

## Results

### Selection of HIV-1 Strains Resistant to Inhibition by the Overexpressed C-Terminal Fragment of LEDGF/p75

Overexpression of LEDGF/p75 truncation mutants containing the IBD severely inhibits viral replication [19]. Figure S1A represents a hypothetical scheme of HIV-1 integration in wild-type (WT) MT-4 cells and MT-4 cells overexpressing the LEDGF/p75 truncation mutant eGFP-Δ325 (MT-4 eGFP-Δ325). The fusion protein eGFP-Δ325 consists of the C-terminal fragment of LEDGF/p75 (amino acids 325 to 530) fused to the C terminus of eGFP. In WT MT-4 cells, the viral IN associates with LEDGF/p75 and is tethered to the chromatin, facilitating the integration of the viral cDNA in the host genome. Upon overexpression of eGFP-Δ325, the eGFP-Δ325 fusion competes with endogenous LEDGF/p75 for interaction with IN and thereby inhibits viral integration. Note that it is possible that recruitment of LEDGF/p75 versus Δ325 does not take place in the nucleus but may already occur in the cytoplasm [6]. Figure S1B displays replication of WT virus in MT-4 eGFP-Δ325 and MT-4 eGFP-Δ325 D366A cells. The control cells overexpress the D366A mutant of eGFP-Δ325 known to be defective for interaction with HIV-1 IN [20]. While HIV-1 replication is readily detected in MT-4 eGFP-Δ325 D366A mutant cell lines, HIV-1 replication is inhibited more than 20-fold 4 d postinfection in the MT-4 eGFP-Δ325 cells. The virus breaking through at 9 d postinfection is not resistant to the inhibitory effect of eGFP-Δ325 (unpublished data); limited HIV replication is allowed in cells where the eGFP-Δ325 fusion protein is competing for LEDGF/p75. Moreover, no mutations were detected in the *IN* gene of this virus (unpublished data). Therefore, we started to select resistant strains by repeated passaging of HIV-1 (NL4.3) in MT-4 eGFP-Δ325 cells. As a control, virus was passaged in parallel in MT-4 eGFP-Δ325 D366A cells. By reducing the volume of the inoculum (i.e., decreasing the multiplicity of infection [MOI]), selective pressure was progressively increased during the experiment.

**Figure 1:**
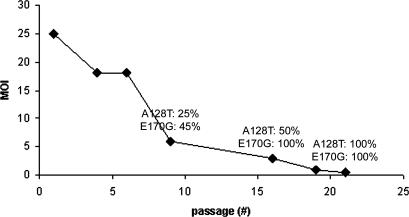
Progressive Accumulation of Mutations in the IN of the HIV-1 Strain Passaged in MT-4 eGFP-Δ325 Cells DNA extracts from HIV-1 (NL4.3) infected MT-4 eGFP-Δ325 cells were made at different passages and a genotypic analysis was performed on the *IN* gene of these strains. The progressive accumulation of mutations in IN is depicted. Amino acid substitution relative to the parental WT HIV-1 (NL4.3) amino acid sequence is shown.

Figure 1 shows the progressive increase in mutation frequency in the IN coding region while increasing selective pressure (decreasing MOI). Selection up to 21 passages (#21) in MT-4 eGFP-Δ325 cells resulted in a virus population with 100% of the strains containing both the A128T and the E170G mutation in the IN coding region. No mutations were identified in the IN coding region of the control virus passaged 21 times on the MT-4 eGFP-Δ325 D366A cells. To rule out cell type dependence, serial passaging of virus was also done in HeLaP4 eGFP-IBD Tet/off and HeLa P4 eGFP-Δ325 Tet/off cells (unpublished data) [19]. These selection experiments identified the mutations A128T, W131G, K156N, and G163E that mapped to the same regions in the *IN* gene. Since MT-4 cells better resemble natural host cells of HIV-1 infection, subsequent experiments focused only on the specific IN mutations found by selection in MT-4 eGFP-Δ325 cells. After 21 passages (#21), two mutations, V38A and S113N, were also found in the gp120 encoding regions of the selected virus population; however, they were present in only 25% and 50% of the virus population, respectively. Moreover, these mutations were also detected in the *gp120* gene of the virus population passaged on MT-4 eGFP-Δ325 D366A cells, although in a lower percentage of the virus population. These mutations likely represent adaptation of the virus to cell culture and infection conditions.

### HIV-1 Overcomes Inhibition by eGFP-Δ325 Overexpression

**Figure 2:**
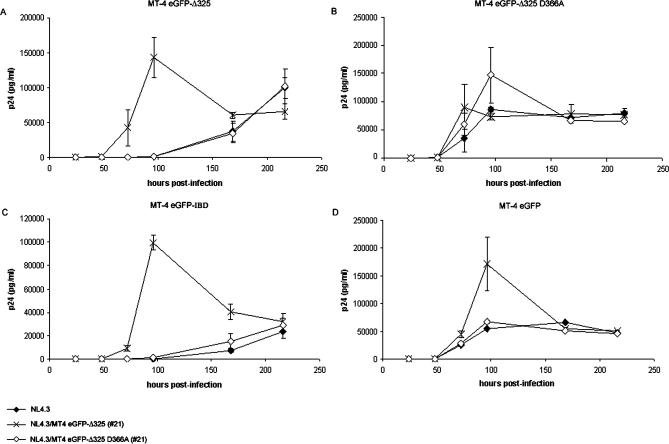
An HIV-1 Strain Is Selected to Replicate in eGFP-Δ325 Overexpression Cells The MT-4 cell lines were infected at an MOI of 0.1 with the following viral strains: WT NL4.3 (filled diamonds), HIV-1 (NL4.3) (crosses) selected for 21 passages in MT-4 eGFP-Δ325 cells (NL4.3/MT-4 eGFP-Δ325 [#21]), and HIV-1 (NL4.3) (open diamonds) selected for 21 passages in MT-4 eGFP-Δ325 D366A cells (NL4.3/MT-4 eGFP-Δ325 D366A [#21]). The viral strains were normalized on p24 content and viral replication was followed by daily measurement of p24 concentration in the supernatant. (A) MT-4 eGFP-Δ325, (B) MT-4 eGFP-Δ325 D366A, (C) MT-4 eGFP-IBD, and (D) MT-4 eGFP cells. All experiments were performed in duplicate. Average ± SD values are shown.

To verify whether the selected HIV-1 strains were indeed less susceptible to the inhibitory effect of the overexpressed LEDGF/p75 C-terminal domain, MT-4 eGFP-Δ325 and MT-4 eGFP-Δ325 D366A cells were infected with the parental HIV-1 strain (NL4.3), the HIV-1 strain selected for 21 passages on MT-4 eGFP-Δ325 cells (NL4.3/MT-4 eGFP-Δ325 [#21]), and the HIV-1 strain selected for 21 passages on MT-4 eGFP-Δ325 D366A cells (NL4.3/MT-4 eGFP-Δ325 D366A [#21]) [19] (Figure 2A and 2B). HIV-1 infection was done at an MOI of 0.1, and the different viral strains were normalized on p24 content. HIV-1 replication was readily detected in control cells overexpressing eGFP (MT-4 eGFP) (Figure 2D) and MT-4 eGFP-Δ325 D366A cells (Figure 2B) for all strains used. As previously shown, no WT viral replication could be detected in MT-4 eGFP Δ325 cells until 96 h postinfection (Figure 2A) [19]. Similar results were obtained for the control virus passaged on MT-4 eGFP-Δ325 D366A cells (NL4.3/MT-4 eGFP-Δ325 D366A [#21]). On the contrary, for the HIV-1 strain selected for 21 passages on MT-4 eGFP-Δ325 cells (NL4.3/MT-4 eGFP-Δ325 [#21]), viral replication in MT-4 eGFP-Δ325 cells was evident from the onset of infection. Identical results were obtained in MT-4 eGFP-IBD cells overexpressing a fusion protein of eGFP to the IBD of LEDGF/p75 [19] (Figure 2C).

### Both A128T and E170G *IN* Mutations Are Necessary and Together Sufficient for Viral Rescue

**Figure 3:**
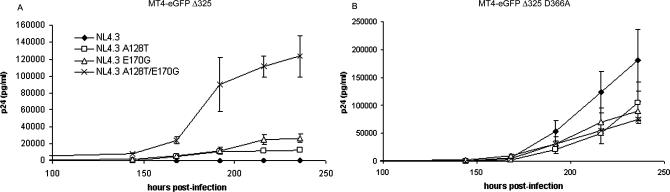
Susceptibility of Mutant Viral Clones to eGFP-Δ325 Overexpression Viral clones that contain either one or both selected mutations were engineered. MT-4 eGFP-Δ325 (A) and MT-4 eGFP-Δ325 D366A cells (B) were infected at an MOI of 0.5 with the following viral clones: WT NL4.3 (diamonds), NL4.3 A128T (boxes), NL4.3 E170G (triangles), or NL4.3 A128T/E170G (crosses). The different viral clones were normalized on p24 content and viral replication was followed by daily measurement of p24 concentration in the supernatant. All experiments were performed in duplicate. Average ± SD values are shown.

To evaluate the importance of the described mutations for the observed resistant phenotype, these mutations were cloned in the HIV-1 NL4.3 molecular clone (pNL4.3) as single or double mutants (pNL4.3 A128T, pNL4.3 E170G, and pNL4.3 A128T/E170G, respectively). Susceptibility of the molecular clones to eGFP-Δ325 overexpression was determined by studying multiple round HIV-1 replications in MT-4 eGFP-Δ325 (Figure 3A) and in MT-4 eGFP-Δ325 D366A cells (Figure 3B). The experiments were carried out at an MOI of 0.5, and the different viral strains were normalized on p24 content. HIV-1 replication was clearly detected in MT-4 eGFP-Δ325 D366A cells for all strains used, although all mutated strains showed a reduced replication capacity compared with WT NL4.3. Whereas the double mutant NL4.3 A128T/E170G rescued HIV-1 replication in MT-4 eGFP-Δ325 cells, the single mutants NL4.3 A128T and NL4.3 E170G could only partially restore viral replication. Apparently, both *IN* mutations are necessary and together sufficient for the strong rescue phenotype of the selected HIV-1 strain in MT-4 eGFP-Δ325 cells.

### Resistance to eGFP-Δ325 Overexpression Is Accompanied by Reduced Viral Replication Kinetics and Reduced Enzymatic Activity of IN

**Figure 4:**
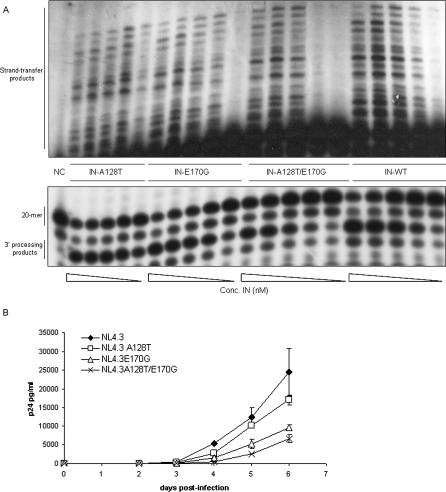
Effect of the IN Mutations on Enzymatic Activity and Replication Kinetics (A) Activities of recombinant IN were tested in an oligonucleotide-based assay. Activity of a 2-fold dilution series of each enzyme was determined, starting at a concentration of 2 μM. Reaction products were separated in a denaturating urea gel and visualized with a PhosphorImager. The enzymatic activity was determined by measuring strand transfer products for the single mutants IN-A128T and IN-E170G and the double mutant IN-A128T/E170G in comparison with WT-IN. Unprocessed substrate was run as a control (lane 1: NC). (B) PBLs were inoculated with WT or mutant viral clones at an MOI of 0.01: WT NL4.3 (diamonds), NL4.3 A128T (boxes), NL4.3 E170G (triangles), or NL4.3 A128T/E170G (crosses). Culture supernatants were collected daily and p24 antigen production was monitored. Experiments were done in duplicate. Average ± SD values are shown.

The mutations detected in the *IN* gene have not been described before. Therefore, we studied the impact of the IN mutations on the enzymatic level in an oligonucleotide-based overall integration assay (Figure 4A). All mutants were enzymatically active, albeit to a lower extent than IN-WT. The IN-A128T enzyme displayed a 3-fold reduction in overall enzymatic activity. The IN-E170G and the IN-A128T/E170G IN mutants both displayed a 2-fold reduction in overall enzymatic activity compared with IN-WT.

To investigate whether the selected IN mutations affect the viral replication kinetics, the mutant molecular clones were examined for their ability to replicate in peripheral blood lymphocytes (PBLs) (Figure 4B). All mutants showed substantially reduced replication fitness in comparison with WT NL4.3 (Figure 4B). Replication kinetics of the double mutant NL4.3 A128T/E170G and the single mutant NL4.3 E170G were decreased 4-fold relative to the replication of WT NL4.3 as evaluated by p24 measurements. The NL4.3 A128T strain showed a 2-fold difference in replication capacity.

### Virus Resistant to eGFP-Δ325 Overexpression Displays Reduced 2-LTR Circle Formation and Integration

To determine the step at which the replication cycle of eGFP-Δ325 resistant virus is affected, real-time quantitative PCR analysis was performed to quantify the formation of the different DNA species in the cell. To ensure single-round infection, 293T cell lines were infected with vesicular stomatitis virus G protein (VSV-G) pseudotyped virus. DNA was extracted at several time points postinfection. As shown in Figure S2A, none of the mutants affected the accumulation of viral DNA at early time points. On the other hand, the number of 2-LTR circles decreased 4-fold for the NL4.3 A128T, NL4.3 E170G, and NL4.3 A128T/E170G virus compared with WT NL4.3 (Figure S2B). The 2-LTR circles are formed in the nucleus and are an indirect indication for nuclear import of the preintegration complex (PIC). Finally, the integration events in 293T cells were measured by real-time Q-Alu PCR. A 3- to 4-fold reduction in the number of proviruses was revealed for all HIV mutants (Figure S2C).

To analyze the rescue of viral replication by real-time quantitative PCR, 293T eGFP-Δ325 cells and control 293T eGFP-Δ325 D366A cells were infected with WT VSV NL4.3 or VSV NL4.3 A128T/E170G. As shown in Figure S2D, the accumulation of viral DNA at early time points was not affected upon expression of eGFP-Δ325. As was previously stated for WT virus, the number of 2-LTR circles increased more than 10-fold in the 293T eGFP-Δ325 cells [19] (Figure S2E). However, this increase was significantly lower when the cells were infected with NL4.3 A128T/E170G virus. Integration could not be measured by real-time Q-Alu PCR, which makes use of an LTR primer that anneals to the lentiviral vector sequences used to make these cell lines. Therefore, infected cells were passaged at least four times (six cell divisions) to dilute nonintegrated DNA, ensuring that only integrated copies of viral DNA remained. The absence of 2-LTR circles at 48 h postinfection (Figure S2E) is an experimental proof for the loss of nonintegrated DNA. Measuring total HIV-1 DNA after four passages pointed to a rescue in the number of proviruses in the 293T eGFP-Δ325 for the NL4.3 A128T/E170G virus in comparison with WT virus (Figure S2F).

### Confocal Imaging Suggests a Decreased Affinity of mRFP-A128T/E170G-IN^s^ for eGFP-Δ325 and LEDGF/p75

Apparently, we selected a virus that can overcome a replication block by a fragment of an essential cofactor, by altering the interaction between IN and the fragment. Experiments were designed to directly demonstrate that the IN mutations affect the interaction with (the C-terminal fragment of) LEDGF/p75. First, confocal imaging was used to study the cellular localization of the different mutant INs (Figure S3A). As expected, due to its interaction with endogenous LEDGF/p75, a transiently expressed monomeric red fluorescent protein-WT-IN^s^ (mRFP-IN^s^) fusion protein localized to the nucleus of WT HeLaP4 cells (Figure S3A) [21–26]. Identical results were obtained for mRFP-A128T-IN^s^. However, mRFP-E170G-IN^s^ and mRFP-A128T/E170G-IN^s^ were more dispersed throughout the cell, although some residual nuclear localization could be observed. This suggests a reduced affinity of the mRFP-A128T/E170G-IN^s^ for LEDGF/p75. Indeed, upon overexpression of eGFP-LEDGF/p75, a remarkable relocalization of the mutant A128T/E170G IN to the nucleus was observed (Figure S3C), which suggests that high levels of LEDGF/p75 can overcome the loss in affinity. As previously described, an explicit cytoplasmic relocalization of both the WT mRFP-IN^s^ fusion protein and eGFP-Δ325 is observed in HeLaP4 eGFP-Δ325 cells (Figure S3B) [19]. Similar results were obtained with mRFP-A128T-IN^s^. But upon overexpression of E170G-IN^s^ or mRFP-A128T/E170G-IN^s^ in HeLaP4 eGFP-Δ325 cells, cytoplasmic relocalisation was less explicit. In the control HeLaP4 eGFP-Δ325 D366A cells, expressing interaction-defective eGFP-Δ325 (Figure S3B), the A128T/E170G fusion protein was also present throughout the cell, reflecting the loss of interaction. These data suggest a reduced affinity of mRFP-A128T/E170G-IN^s^ for the eGFP-Δ325 fusion protein.

### Characterization of the Interaction between the Mutant INs and LEDGF/p75 or eGFP-Δ325

**Figure 5:**
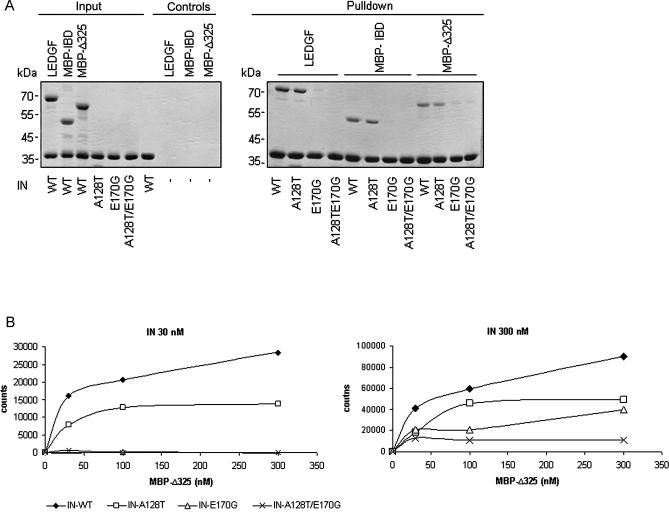
Resistance to eGFP-Δ325 Overexpression Is Accompanied by Reduced In Vitro Binding to LEDGF/p75 (A) His_6_-tag IN mediated pull-down of LEDGF/p75, MBP-IBD, and MBP-Δ325. Recombinant LEDGF/p75, MBP-IBD, or MBP-Δ325 was incubated with His_6_-tagged HIV-1 IN, and complexes were recovered using Ni^2+^-NTA beads. Proteins were separated via a 12.5% SDS-PAGE gel and detected using Coomassie Blue staining. Molecular weight markers are indicated on the left side of each gel. Pull-down controls, which represent pull-downs of single proteins, are shown in the left gel, next to the input of each protein. The right gel displays the recovered proteins after pull-down. (B) Interactions between His_6_-tagged IN and MBP-Δ325 were determined using AlphaScreen technology. Two different concentrations of IN (30 nM or 300 nM) were incubated with various amounts of MBP-Δ325. As a negative control, 300 nM MBP-Δ325 was incubated without IN. Ni-chelate–coated acceptor beads and anti-MBP–coated donor beads were added to bind IN or MBP-Δ325, respectively. IN-MBP-Δ325 interaction was measured with the AlphaScreen method. Experiments were performed twice. A representative experiment is shown.

Confocal imaging suggests a decreased affinity of mRFP-A128T/E170G-IN for eGFP-Δ325 and LEDGF/p75. To further characterize these interactions, we investigated the ability of the mutant INs to interact with LEDGF/p75, MBP-IBD, and MBP-Δ325 using an in vitro pull-down assay (Figure 5A). Both C-terminal His-tagged WT IN^s^ (IN-WT) and IN containing the A128T mutation (IN-A128T) were able to pull down LEDGF/p75, MBP-IBD, and MBP-Δ325. On the contrary, almost no interaction was detected with the E170G or A128T/E170G double mutation. The same results were obtained when N-terminal WT and mutant His-tagged INs for pull-down were used (unpublished data). The highly reduced interaction of MBP-Δ325 with IN-E170G and IN-A128T/E170G was analyzed in more detail with different IN and MBP-Δ325 concentrations using AlphaScreen technology (Figure 5B). In this assay, C-terminal His_6_-tagged IN was added to different concentrations of MBP-Δ325. Ni-chelate–coated acceptor beads and anti-MBP–coated donor beads were added to bind IN or MBP-Δ325, respectively. Binding of the molecules to the beads results in an energy transfer from one bead to the other, producing a fluorescent signal. Representative interaction spectra are shown in Figure 5B. Background fluorescence was subtracted from the fluorescence signal of the samples. At an IN concentration of 30 nM, the fluorescent signal of the IN-WT and IN-A128T enzymes increased upon increasing MBP-Δ325 concentrations (from 30 to 300 nM) until a plateau was reached. No fluorescent signal above background could be detected for the IN-E170G and the IN-A128T/E170G mutants. When a higher amount of IN (300 nM) was incubated with increasing MBP-Δ325 concentrations (from 30 to 300 nM), fluorescent signal could also be detected for the IN-E170G and the IN-A128T/E170G mutants. Still, interaction of IN-A128T/E170G to MBP-Δ325 (300 nM) was reduced 8-fold compared with IN-WT. Binding of IN-A128T and IN-E170G to MBP-Δ325 was reduced 2-fold and 3-fold, respectively. These results clearly indicate that the affinity of the double mutant IN-A128T/E170G to MBP-Δ325 is highly reduced. However, at high concentrations of both proteins, in vitro binding remains possible.

**Figure 6:**
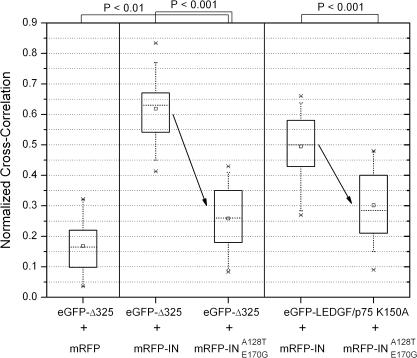
In Vivo Interaction of WT and Mutant HIV-1 IN with LEDGF/p75 and eGFP-Δ325 HeLaP4 eGFP-Δ325 or HeLaP4 cells overexpressing eGFP-LEDGF/p75(K150A) in the cytoplasm were transfected with plasmids encoding WT or double mutant A128T/E170G mRFP-IN^s^. As a negative control, cells were transfected with an mRFP expression plasmid. Interaction between eGFP-Δ325 or eGFP-LEDGF/p75(K150A) with WT mRFP-IN^s^ or A128T/E170G mRFP-IN^s^ was detected by measuring the simultaneous diffusion of the differentially labeled proteins using FCCS. The interaction is presented as the relative extent of cross-correlation (data are shown in box plots with SD for 30 measurements). A two-sample *t*-test was performed to prove that the different samples were drawn from different populations. The two samples are statistically different if *p* < 0.01. Since cross-correlation is concentration dependent, WT IN was compared with double mutant IN for binding to eGFP-Δ325 and for binding to eGFP-LEDGF/p75. The respective reduction in affinity is represented by the arrow, and the accompanying *p*-values are shown at the top of the figure.

To further characterize these interactions in vivo, we applied fluorescence cross-correlation spectroscopy (FCCS) in HeLa-P4 derived cell lines. This method allows the quantification of the interaction of two fluorescently labeled proteins in cellulo by measuring simultaneous diffusion. Because the diffusion of the complex mRFP-IN^s^ and eGFP-LEDGF/p75 is strongly reduced in the nucleus—most probably due to interactions with chromatin—we could not quantify interactions in the nucleus. Therefore, the K150A NLS mutation was introduced in LEDGF/p75 to overcome the nuclear localization [27], and measurements were done in the cytoplasm of the cell. HeLaP4 eGFP-Δ325 or HeLaP4 cells overexpressing eGFP-LEDGF/p75(K150A) were transfected with plasmids encoding WT or double mutant mRFP-IN^s^. As a negative control, cells were transfected with an mRFP expression plasmid. Compared to the negative control, cross-correlation of eGFP-Δ325 or eGFP-LEDGF/p75(K150A) with WT mRFP-IN^s^ was readily detected (*p* < 0.01), indicating that both proteins interact with WT IN in the cytoplasm of the living cell (Figure 6). The binding of WT and double mutant IN to eGFP-Δ325 was compared as well as the interaction of WT and double mutant IN with eGFP-LEDGF/p75. The mutations affected both interactions significantly (*p* < 0.001). The cross-correlation signal for the interaction of A128T/E170G mRFP-IN^s^ with eGFP-Δ325 was more affected than that for interaction with full-length LEDGF/p75, suggesting that the mutations in viral IN differentiate between Δ325 and full-length LEDGF/p75.

A crystal structure of the dimeric catalytic core domain (CCD) of HIV-1 IN complexed to the IBD of LEDGF/p75 was previously reported [28]. A128 is part of the α3 helix in the IN-CCD, which forms a hydrophobic patch that accommodates the side chains of the LEDGF/p75 residues I365, F406, and V408 [28]. E170 is part of the so-called α4/5 connector, a six-residue connector linking helices α4 and α5 of the second IN chain (residues 166 through 171). These two regions were reported to be the main structural features of IN recognized by LEDGF/p75. Biomolecular modeling was performed to predict the influence of the IN mutations on the interaction of IN and LEDGF/p75 (Figure S4A and S4B). Substituting alanine for threonine at position 128, and performing a structure energy minimization, resulted in a slightly decreased binding affinity of IN for IBD, with an increase in binding energy from −68.02 to −61.03 kcal/mol. Apparently, the bulky side chain of threonine causes sterical hindrance, thereby forcing the I368 residue of the IBD to adapt a different conformation. This occurs without a drastic influence on the conformation of residues in the LEDGF/p75–IN binding interface. Substituting glutamic acid for glycine at position 170 has a more drastic effect. This mutation results in a different conformation of the loop disrupting two interactions. First, the double hydrogen bond between the hydrogens of the loop backbone at residues E170 and H171 and D366, a critical interacting residue on LEDGF/p75, is broken. Second, the electrostatic interaction of E170 (IN) with K364 (LEDGF/p75) is abolished. These effects cause a significant decrease in binding affinity, with an increase in predicted binding energy from −68.02 to −56.38 kcal/mol. When both IN mutations are present, the overall binding energy for the IBD–IN complex is increased from −68.02 to −53.10 kcal/mol.

### Virus Resistant to eGFP-Δ325 Overexpression Is Dependent on LEDGF/p75 for Replication

**Figure 7:**
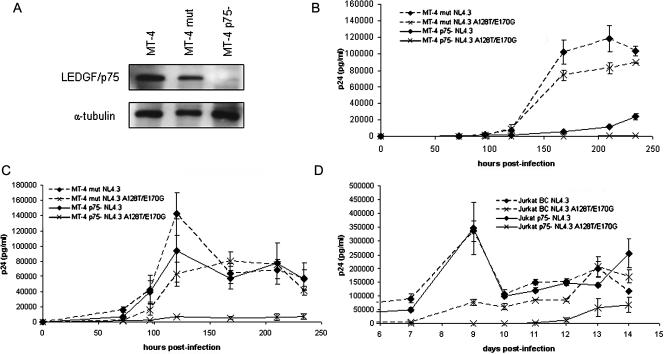
Virus Resistant to eGFP-Δ325 Overexpression Is Dependent on LEDGF/p75 for Replication (A) Western blot analysis of LEDGF/p75 expression levels in MT-4 cells (lane 1) and stable MT-4 knockdown cell lines (MT-4 p75−) (lane 3). As a control, MT-4 cells expressing a mismatch shRNA against LEDGF/p75 were made (MT-4 mut) (lane 2). Equal loading of gels was controlled by α-tubulin detection. (B and C) MT-4 p75− and MT-4 mut cells were infected at an MOI of 0.5 (B) or 2.5 (C) with the following viral clones: WT NL4.3 (diamonds) or NL4.3 A128T/E170G (crosses). (D) Jurkat cells encoding a shRNA directed against LEDGF/p75 (Jurkat p75− cells) and BC Jurkat cells were infected at an MOI of 0.5 with WT NL4.3 (diamonds) or NL4.3 A128T/E170G (crosses). The different viral strains were normalized on p24 content, and viral replication was followed by daily harvesting of the supernatant and measurement of p24 concentration. Experiments were run in duplicate. Average ± SD values are shown.

How does HIV overcome the replication block induced by the C-terminal fragment of its cofactor? FCCS measurements suggest that by altering the affinity of IN for the cofactor, HIV-1 may be capable to discriminate between endogenous LEDGF/p75 and the inhibiting C-terminal fragment (eGFP-Δ325). Alternatively, HIV-1 that has become deficient for interaction with LEDGF/p75 may replicate independently from LEDGF/p75, by employing another cofactor. The latter explanation has been put forward recently after analysis of integration sites in LEDGF/p75 knockdown cells [14]. LEDGF/p75 knockdown only resulted in a modest shift in integration site selection. To address this question, replication of the virus resistant to eGFP-Δ325 overexpression was followed in LEDGF/p75 knockdown cells. Stable polyclonal MT-4 LEDGF/p75 knockdown cells were generated using lentiviral vectors encoding shRNA targeting LEDGF/p75 (MT-4 p75−). As a control, four mutations were introduced in the short hairpin against LEDGF/p75, and MT-4 cells were generated (MT-4 mut). Efficiency of knockdown was verified by Western blotting (Figure 7A). Infection of these cell lines with WT NL4.3 at an MOI of 0.5 resulted in a 5- to 10-fold reduction in viral replication as measured by p24 antigen production in the supernatant (Figure 7B). In the control cells encoding the mutant hairpin (MT-4 mut), no reduction of HIV-1 replication was observed. No breakthrough of the mutant NL4.3 A128T/E170G virus was detected up to 10 d postinfection in MT-4 p75− cells (MOI 0.5) (Figure 7B). Moreover, viral replication of the NL4.3 A128T/E170G virus was inhibited 10-fold more than that of WT virus in MT-4 p75− cells. In the control MT-4 mut cells, both strains showed equivalent viral replication. The inhibitory effect on the WT virus in the MT-4 p75− cells could be overcome by using a higher MOI (MOI 2.5) (Figure 7C). However, at the same MOI, NL4.3 A128T/E170G replication remained 10-fold lower. The same experiment was performed in the LEDGF/p75 knockdown Jurkat cells p75(−) Cl 2 (Jurkat p75−) and p75(−) Cl 2 backcomplemented (BC) (Jurkat BC) cells that were originally reported by Llano et al. [6]. Llano and coworkers did not detect any reduction in viral replication in these knockdown cells compared with the BC control cells. In agreement, we could not detect any inhibition of replication of WT NL4.3 in these cells (Figure 7D). However, replication levels of A128T/E170G virus were 10-fold lower in the Jurkat p75− cells, whereas no significant reduction of HIV-1 replication was observed in the BC Jurkat cells for any of the strains used. We reason that replication of the mutant virus should not be inhibited in cells with highly reduced levels of LEDGF/p75, if this replication occurs independently of LEDGF/p75. To the contrary, our experiments suggest that the virus resistant to eGFP-Δ325 overexpression has a reduced affinity for LEDGF/p75, making it more vulnerable to reduction of LEDGF/p75 levels. It is clear that LEDGF/p75 is still employed by the virus for viral replication in eGFP-Δ325 overexpressing cells. Our data are thus at odds with the existence of another cofactor that can substitute for LEDGF/p75.

In line with these results and to further strengthen our model that the A128T/E170 virus is still capable of interacting with LEDGF/p75 in vivo, we speculated that poor replication of the double mutant virus in WT cells could be rescued by overexpression of LEDGF/p75. For this purpose, MT-4 cells stably overexpressing LEDGF/p75 and MT-4 cells stably overexpressing eGFP were generated. Overexpression was verified by Western blot analysis (Figure S5A). These cell lines were used to compare replication of WT NL4.3 virus and A128T/E170G NL4.3 virus (Figure S5B). In the control MT-4 eGFP cells, replication kinetics of the double mutant NL4.3 A128T/E170G was decreased 2-fold in comparison to the WT HIV-1 NL4.3. However, in MT-4 cells overexpressing LEDGF/p75, replication of both virus strains was similar. This experiment again suggests that poor replication of the IN mutants in WT cells is due to reduced affinity for LEDGF/p75 and that the A128T/E170 IN mutant virus still exploits LEDGF/p75 in vivo*.*

## Discussion

We have previously characterized LEDGF/p75 mutants containing the IBD but lacking the chromosome attachment site (eGFP-Δ325 and eGFP-IBD) that strongly impair HIV-1 replication by competing for the interaction with IN [19]. We have now selected HIV-1 strains resistant to such a truncation mutant of LEDGF/p75 (eGFP-Δ325) (Figure 2). Two mutations were detected in the IN coding region at key positions in the LEDGF/p75–IN interface (A128T and E170G) (Figure 1). This corroborates that the specificity of the observed inhibition in viral replication in MT-4 eGFP-Δ325 cells is unambiguously based on the direct interaction of the overexpressed Δ325 fragments with IN. The available crystallographic data on the IN-IBD interface [28] were recently questioned after an in vitro mutagenesis study that failed to observe a strict correlation between the interaction of IN with LEDGF/p75 and the effect on HIV-1 replication [29]. Detection of the IN mutations A128T and E170G at key positions after resistance selection provides in vivo confirmation for the previously reported structure. As biomolecular modelling suggests, mutating these residues compromises the local structure of the IBD−IN CCD interface, affecting the interaction with LEDGF/p75 truncation mutants (Figure S4). Our data provide a striking example of the power of viral molecular evolution. Not only is the approach apparently more relevant than in vitro mutagenesis, a virus appears to discriminate between a fragment of a critical cofactor and the full-length cofactor itself.

To overcome inhibition by Δ325, mutations in the IN–IBD interface are selected. These mutations cripple the virus considerably (Figure 4). It has been demonstrated before that mutations associated with antiviral resistance to diketo acids reduce the enzymatic activity of IN [30,31]. The conservation of the *IN* gene compared with other viral genes [32] suggests a tight restriction in amino acid flexibility. Still, our results show that viral replication is more affected than enzymatic activity. The most plausible explanation for the observed phenotype is that by altering the affinity for the inhibiting C-terminal fragment, interaction with the critical cofactor LEDGF/p75 is also jeopardized. Indeed, poor replication of the double mutant virus in WT cells could be rescued by overexpression of LEDGF/p75 (Figure S5B). Although HIV strains have been selected to grow independently of restriction factors [33,34], this is to our knowledge the first example of selection of a virus that is partially defective for binding to its cellular cofactor. Although humans carrying a 32–base pair deletion of the *CCR5* gene are resistant to HIV-1 infection, no R5 virus has yet been isolated that overcomes this inhibitory effect; infection is only possible via T-tropic virus that uses the CXCR4 coreceptor [35].

The reduced replication kinetics of the mutated virus were further analyzed by quantitative PCR analysis of 293T cells infected with VSV-G pseudotyped A128T/E170G virus. We detected a 4-fold decrease in the number of 2-LTR circles and in integrated provirus (Figure S2). In previous experiments in which integration was impaired due to partial depletion of LEDGF/p75 [16,17] or to the presence of competing C-terminal fragments [19], the amount of 2-LTR circles was either constant or increased. The reduction in 2-LTR circles might indicate that the mutations in IN affect nuclear import of the PIC or the formation of 2-LTR circles. We cannot exclude that reduced interaction with LEDGF/p75 is responsible for this phenotype. Our previous finding that LEDGF/p75 drastically increases the affinity of lentiviral IN for DNA may imply a role of LEDGF/p75 in the formation of the complex between IN and the viral DNA. This is the first experimental evidence suggesting that IN-LEDGF/p75 interaction might affect steps other than chromosomal tethering/targeting. The documented presence of LEDGF/p75 in the cytoplasmatic PIC is consistent with this concept [6].

Analysis of DNA formation by the mutant virus in eGFP-Δ325 overexpressing 293T cells revealed that integration of the resistant virus in the 293T eGFP-Δ325 cells was restored (Figure S2F). Whereas the number of 2-LTR circles produced by WT virus increased more than 10-fold in the 293T eGFP-Δ325 cells (Figure S2E) as a result of the defect in integration (Figure S2F) [19], the number of 2-LTR circles returned to almost normal levels when the cells were infected with A128T/E170G virus. These data confirm that the C-terminal fragments specifically block the integration and/or targeting step.

What is the mechanism underlying the resistant phenotype observed? Our in vitro data (Figure 5) and in vivo confocal microscopy analysis (Figure S3) both reveal experimentally that the mutations that reduce interaction with eGFP-Δ325 also decrease interaction with full-length LEDGF/p75. The available structural data support this result. The fact that A128T/E170G IN displays a reduced affinity for full-length LEDGF/p75 seems to suggest at first sight that HIV can replicate without LEDGF/p75 perhaps using an alternative cofactor. In fact, because of the subtle alterations in target site selection after LEDGF/p75 depletion, the existence of such a secondary targeting cofactor has been proposed [14]. However, replication of the resistant virus in LEDGF/p75 knockdown MT-4 cells was inhibited 10-fold more than that of WT virus (Figure 7B). Moreover, replication of A128T/E170G virus was also severely impaired in the Jurkat p75− cells, which support normal replication of WT virus [6] (Figure 7D). It follows that LEDGF/p75 is still employed by the resistant virus that is capable to grow in eGFP-Δ325 overexpression cells. In fact, poor replication of the double mutant virus in WT cells is rescued by overexpression of LEDGF/p75 (Figure S5B), corroborating that the A128T/E170 IN mutant virus still exploits LEDGF/p75 in vivo*.* Although we cannot formally exclude the remote possibility that both mutations also affect binding of the alternative cofactor, its existence has become unlikely. Reduction of the affinity of IN for LEDGF/p75 results in a clear phenotype even in cells with partial depletion of LEDGF/p75. Hence, the failure by Llano et al. [6] to detect a replication deficit in Jurkat p75− is due to an insufficient knockdown, as proposed [1,16]. Apparently, reduced interaction with LEDGF/p75 is preferred rather than ineffective targeting to the chromatin in the absence of LEDGF/p75.

Replication of A128T/E170G virus in MT-4 cells overexpressing LEDGF/p75 truncation mutants suggests that its affinity for eGFP-Δ325 is more reduced than for LEDGF/p75, taking into account that eGFP-Δ325 is in excess of LEDGF/p75 (unpublished data). Differential impact of the A128T/E170G mutation on interaction of recombinant IN with full-length LEDGF/p75 or the C-terminal fragment could not be documented in vitro by pull-down (Figure 5A) or AlphaScreen technology (unpublished data). Probably, these assays do not reflect the in vivo situation. In vivo FCCS experiments, however, revealed that mutant IN has a different affinity for the cofactor and the inhibiting C-terminal fragment (Figure 6). Domains in LEDGF/p75 other than the C-terminus may contribute to the interaction with IN. Alternatively, the fusion of Δ325 to eGFP may also aid A128T/E170G IN to preferentially bind to LEDGF/p75.

The importance of LEDGF/p75 for HIV replication has been demonstrated over time using mutagenesis [4], RNA interference [16,17], or overexpression of truncation mutants [19]. However, none of these experiments could exclude the existence of other cofactors capable of tethering the PIC to the chromosomes. Our data indicate that HIV is incapable to circumvent the LEDGF/p75 function during integration. Our approach may prove valuable in validating other cellular cofactors of virus replication. Demonstration of the biological relevance of the available LEDGF/p75–IN interface, the impaired replication kinetics upon resistance selection to an interaction inhibitor, and the exclusive role in HIV integration all support the attraction of LEDGF/p75 as an antiviral target.

## Materials and Methods

### Plasmids and viral vectors.

The construction of the lentiviral vector transfer plasmids encoding shRNA targeting LEDGF/p75 was described previously (unpublished data). The construct pmRFP-IN^s^ was used for overexpression of mRFP-IN^s^ in HeLaP4 cells [19], and the construct peGFP-p75 was used to overexpress eGFP-tagged human LEDGF/p75 in HeLaP4 cells [12]. For bacterial expression of nontagged LEDGF/p75, C-terminal His_6_-tagged HIV-1 IN and N-terminal His_6_-tagged HIV-1 IN, the plasmids pCPnat75 [12], pKB-IN6H [12], and pRP1012 [36] were used, respectively. To generate the A128T or E170G single mutants and double mutants, site-directed mutagenesis was performed using the Kirsch and Joly method [37]. The presence of the expected mutations was confirmed by DNA sequencing of the entire *IN* coding region to ensure no other mutations were present in the molecular clone. To construct the lentiviral transfer plasmids pCombi_LEDGF_IRES_Puro, eGFP was removed from the plasmid pCombi_eGFP_IRES_Puro [19] by BamHI-SpeI digestion and replaced by BamHI-LEDGF-XbaI. This fragment was obtained by PCR amplification using the primers LEDGF-forw (5′-GGC GGG ATC CAG ACA CCA TGA CTC GCG ATT TCA AAC C-3′) and LEDGF-rev (5′-CAG GTC TAG ACT AGT TAT CTA GTG TAG AAT CCT TC-3′) using pEF1-LEDGF-Back [6] as a template. Subsequently, the resulting products were digested with BamHI and SpeI. Production of lentiviral vectors was performed as described previously by Geraerts et al. [38].

### Cells.

MT-4 cells [39], 293T cells, HeLaP4 cells, Jurkat cells, and PBLs were grown in a humidified atmosphere with 5% CO_2_ at 37 °C. The 293T cells were obtained from O. Danos (Génethon, Evry, France) and grown in DMEM (GIBCO BRL, Merelbeke, Belgium, http://www.invitrogen.com) supplemented with 10% heat-inactivated fetal calf serum (FCS; Harlan Sera-Lab, http://www.harlaneurope.com, 2 mM glutamine (GIBCO BRL), 100 U/ml penicillin (GIBCO BRL), and 100 μg/ml streptomycin (GIBCO BRL). HeLaP4 cells, obtained from the National Institutes of Health Reagent program, were grown in DMEM containing 10% FCS, 100 μg/ml streptomycin, 100 U/ml penicillin, and 0.5 mg/ml geneticin (GIBCO BRL). MT-4 cells and Jurkat cells were maintained in RPMI 1640 (GIBCO BRL) supplemented with 10% heat-inactivated FCS, 2 mM l-glutamine, 0.1% sodium bicarbonate (GIBCO BRL), 100 U/ml penicillin, and 100 μg/ml streptomycin. Medium for maintenance of Jurkat cells additionally contained 0.1% sodium pyruvate (100 mM) (GIBCO BRL). Peripheral blood was obtained by venipuncture from HIV-seronegative healthy donors. PBLs were isolated by centrifugation on gradient medium (Lymphoprep; Axis-Shield, Oslo, Norway), washed twice in PBS, and resuspended in RPMI 1640. PBLs were maintained in RPMI 1640 supplemented with 15% heat-inactivated FCS, 2 mM l-glutamine, 0.1% sodium bicarbonate, and 0.5 mg/ml gentamycin (GIBCO BRL). Cells were stimulated 3 d prior to use with 5 U/ml interleukin-2 (Roche Applied Science, http://www.roche-applied-science.com) and 2 μg/ml phytohemagglutinin (PHA) (Sigma-Aldrich, http://www.sigmaaldrich.com). During experiments, PBL medium contained 10 U/ml interleukin-2. Generation and selection of MT-4 eGFP-Δ325, MT-4 eGFP-Δ325 D366A, MT-4 eGFP-IBD, MT-4 eGFP, and its HeLa P4 and 293T equivalents were described previously [19]. Jurkat cells p75(−) Cl 2 and Jurkat cells p75(−)Cl 2 BC were obtained from E. M. Poeschla (Rochester, Minnesota, United States) [13]. Generation and selection of stable MT-4 LEDGF/p75 knockdown cells were done using lentiviral vector transfer plasmids encoding an shRNA targeting LEDGF/p75 driven by an mU6 promotor and a CMV-driven eGFP-ZEOCIN fusion gene (unpublished data). MT-4 cells (1 × 10^5^ ) were transduced at an MOI of 1 and selected with 200 μg/ml zeocin (Invitrogen). Finally, the 10% of the highest MT-4shp75 eGFP expressor cells were sorted, until a total of 10^5^ cells was reached, using the FACSVantage (BD Biosciences, http://www.bdbiosciences.com) [19]. A similar approach was used for the generation of the MT-4 mut cell lines. Four mutations were introduced in the short hairpin that was expressed from the transfer plasmid of the lentivral vector used. MT-4 cells stably overexpressing LEDGF/p75 (MT-4 LEDGF/p75) or eGFP (MT-4 eGFP) were generated by transducing MT-4 cells with Combi_LEDGF_IRES_Puro vector or Combi_eGFP_IRES_Puro vector, respectively. At 24 h prior to transduction, cells were seeded in a 96-well plate at a density of 100,000 cells/well. After overnight transduction at 37 °C in a 5% CO_2_ humidified atmosphere, the medium was replaced. Five days posttransduction, selection with puromycin (0.25 μg/ml) was initiated. At different time points during selection, LEDGF/p75 expression was analyzed by Western blotting.

### Virus strains.

The HIV-1 molecular clone pNL4.3 [40] was obtained through the AIDS Research and Reference Reagent Program, Division of AIDS, National Institute of Allergy and Infectious Diseases, National Institutes of Health, contributed by Dr. Malcolm Martin (Bethesda, Maryland, United States). To generate the A128T, E170G, and A128T/E170G IN mutated viruses, site-directed mutagenesis was performed using the Kirsch and Joly method [37]. The presence of the expected mutations was confirmed by DNA sequencing of the entire *IN* coding region. Virus productions were performed as described by De Rijck et al. [19].

### Selection of resistance.

The resistance selection of HIV-1 (NL4.3) against overexpression of the C-terminal part of LEDGF/p75 was initiated at a high MOI (25) in MT-4 eGFP-Δ325 cells. Simultaneously, as a control, virus was also passaged in MT-4 eGFP-Δ325 D366A cells. Every 3 to 4 d, the MT-4 cell culture was monitored for the appearance of HIV-induced cytopathic effect (CPE). When CPE was observed, the cell-free culture supernatant was used to infect fresh, uninfected MT-4 eGFP-Δ325 or MT-4 eGFP-Δ325 D366A cells. To increase selective pressure, the volume of supernatant transferred was progressively decreased during the experiment from 1 ml to 50 μl for 2 × 10^5^ cells cultured in 2 ml of medium.

### PCR amplification and sequencing of the coding regions of IN.

Proviral DNA extraction of infected MT-4 eGFP-Δ325 cells was performed using the QIAamp blood kit (Qiagen, http://www.qiagen.com). PCR amplification and sequencing of IN encoding sequences were done as described previously [30]. Mutations present at least 25% of the global virus population can be detected as a mixture with the WT amino acid by means of population sequencing.

### HIV-1 infection.

Infection of MT-4 and Jurkat cells was performed with 1 × 10^6^ cells in 1 ml of medium at an MOI of 0.1, 0.5, or 2.5 for 3 h. Different virus strains were normalized on p24 levels. After 3 h, cells were washed twice with PBS and resuspended in 10 ml of MT-4 medium. HIV-1 replication was monitored by quantifying p24 antigen in the supernatant via ELISA (Alliance HIV-1 p24 ELISA kit; Perkin Elmer, http://www.perkinelmer.com).

### Replication kinetics.

Either 500 or 5,000 TCID_50_ (50% tissue culture infective doses) of each virus was used to infect 5 × 10^5^ PHA-stimulated PBLs (MOI = 0.001 or 0.01). TCID_50_ were determined using the Spearman-Karber method [41]. Culture supernatants were collected every day until day 7, and p24 antigen production was monitored by ELISA (Perkin Elmer).

To study replication kinetics in MT-4 cells, inoculants of various HIV-1 strains containing equal amounts of HIV-1 p24 antigen (10, 5, and 2.5 pg/ml) were added to MT-4 cells (50,000 cells/ml). From 3 d postinfection on, aliquots of cell-free supernatants were taken for p24 level determination.

### Real-time quantitative PCR analysis.

One day prior to transduction, 293T cells were seeded in 24-well plates at approximately 2 × 10^5^ cells per well. Transductions with the VSV-G pseudotyped viruses were carried out at an MOI of 10. Prior to infection, VSV-G virus stocks were treated with RNase-free DNase I (0.2 U/μl) (Roche Applied Sciences). Virus was added to the cells in the presence of DMEM–1% FCS. After 3 h of incubation, medium was replaced by DMEM containing 10% FCS. In each 24-well plate, noninfected 293T cells were incubated in parallel. DNA extractions and quantification of late reverse transcripts, 2-LTR circles, and integrants were done as described earlier [42,43]. Analysis of DNA extracted from lentivirus tranduced cells was done as described earlier [19].

### Laser scanning microscopy.

HeLaP4, HeLaP4 eGFP-Δ325, and HeLaP4 eGFP-Δ325 D366A cells were transfected with pmRFP-IN^s^ and analyzed using a LSM510 unit (Zeiss, Zaventem, Belgium) as described previously [16]. Overexpression of LEDGF/p75 in HeLa P4 cells was accomplished by cotransfection with peGFP-p75 [12].

### FCCS analysis.

FCCS measurements were performed on a commercial LSM510/ConfoCor2 system (Carl Zeiss, Jena, Germany). The 488-nm line of the Ar^+^- laser (AOTF 0.1%, approximately 25 μW) was used to excite eGFP, and the 543-nm line of the HeNe laser (AOTF 7%, approximately 70 μW) was used to excite mRFP1. The excitation light was reflected by a dichroic mirror (HFT 488/543) and focused through a type C-Apochromat ×40/1.2W objective lens. The fluorescence emission light was split by a second dichroic mirror (NFT 570) into two separate beam paths and passed through a 505- to 530-nm bandpass filter for eGFP fluorescence and a 600- to 650-nm bandpass filter for mRFP1 fluorescence. Each confocal pinhole diameter was set to 70 μm. After preparation of the cells in an eight-well chambered coverglass (NUNC A/S, Roskilde, Denmark), laser scanning microscopy was used to search for cells suitable for FCCS. The laser beam was focused at a selected spot, and an FCCS measurement (10 × 20 s) was performed. Auto- and cross-correlation curves were evaluated by a Levenberg-Marquardt nonlinear least-squares fitting to a two-component model, using the OriginPro 7.5 software (OriginLab, Northampton, Massachusetts, United States). The relative cross-correlation is calculated from the following formula: CC_rel._ = G_cross_(0)/G_green_(0), with G_cross_(0) and G_green_(0) being the amplitudes of the cross and green autocorrelation curves, respectively [19].

### Western blotting.

Western blotting was performed as described previously [16].

### Production and purification of recombinant proteins.

Nontagged LEDGF/p75 was produced from the plasmids pCPnat75 in Escherichia coli BL21(DE3) and purified as described previously [12]. The construction of pMBP-IBD, pMBP-Δ325, and pMBP-Δ325 D366A and purification of these fusion proteins from BL21 bacterial cells were described previously [19]. HIV-1 IN enzymes were purified on a nickel-nitrilotriacetic acid column (Qiagen), followed by a phosphocellulose cation exchange column (Whatman International, http://www.whatman.com). The purified recombinant proteins were concentrated by ultrafiltration using Centricon10 (Millipore, http://www.millipore.com) or Vivaspin 15R (Vivascience, http://www.vivascience.com). All IN protein concentrations were measured using the Bradford assay (Bio-Rad, http://www.bio-rad.com). The purified recombinant enzymes were analyzed on SDS-PAGE, supplemented with 5 mM dithiothreitol (DTT) plus 10% glycerol, and frozen at −80 °C.

### Substrate and target DNA used in the enzymatic IN assay.

The HPLC-purified deoxyoligonucleotides, INT1 (5′-TGT GGA AAA TCT CTA GCA GT) and INT2 (5′-ACT GCT AGA GAT TTT CCA CA), corresponding to the U5 end of the HIV-1 LTR, were purchased from Amersham Biosciences (http://www.amersham.com). The oligonucleotide INT1 was purified through a 20% denaturing polyacrylamide/urea gel and was radioactively labeled at the 5′-end using polynucleotide T4 kinase and [γ-^32^P]ATP (Amersham Biosciences). The DNA substrate and target for IN reactions were made by annealing INT1 and INT2. An equimolar mixture of the two oligonucleotides in the presence of 100 mM NaCl was heated shortly at 95 °C and allowed to cool slowly to room temperature.

### The overall integration assay.

The enzymatic integration reactions were carried out as described previously [44–46] with minor modifications [30]. Specific activities of different enzyme preparations were determined in this assay. The extent of overall integration was based on measuring the amounts of strand transfer products of the mutant INs in comparison with WT IN. These data were determined using the OptiQuant Acquisition and Analysis software (Perkin Elmer Corporate).

### His_6_ Tag IN Pull-down Assay.

His_6_ tag IN pull-down assay was adapted from Maertens et al. [12], with minor modifications. Binding of IN to LEDGF/p75, IBD-MBP, and Δ325-MBP was assayed in 25 mM Tris-HCl (pH 7.4), 0.1% Nonidet P-40, and 20 mM imidazole containing 150 mM NaCl, in the presence of 1 mM MgCl_2_ (binding buffer). Then, 6 μg of recombinant C-terminal His_6_-tagged IN was incubated with 40 μl of Ni-NTA-agarose for 10 min at 4 °C. Next, 6 μg of LEDGF/p75 in 200 μl of binding buffer supplemented with 4 μg of BSA was added to the mixture. Following 4-h incubation at 4 °C, the agarose beads were recovered by centrifugation for 2 min at 13,000 rpm at 4 °C and washed several times with 300 μl of binding buffer. Bound proteins were eluted in 20 μl of binding buffer supplemented with 200 mM imidazole and 1% SDS and analyzed by 11% SDS-PAGE followed by staining with Coomassie Blue R-250 [12].

### AlphaScreen.

To confirm the loss of interaction of the mutant INs to the Δ325 fragment, AlphaScreen technology (Perkin Elmer) was performed. In a 384-well plate, 0, 30, 100, or 300 nM of IN was added to 0, 10, 30, or 100 nM MBP-Δ325 at room temperature in assay buffer (25 mM Tris/HCl [pH 7.3], 150 mM NaCl, 1 mM MgCl_2_, 0.01% Tween-20, 0.1% BSA). The mixture was incubated for 90 min at room temperature. For subsequent binding of the proteins to the beads, Ni-chelate–coated acceptor beads and streptavidin-coated donor beads premixed with biotinylated anti-MBP antibody (Vector Laboratories, http://www.vectorlabs.com) were added to a final concentration of 20 μg/ml. All dilutions were done in assay buffer and, if necessary, the final volume of the reaction was adjusted to 25 μl. In order to bind the anti-MBP antibody to the streptavidin-coated donor beads, the antibody was dialyzed overnight against assay buffer without BSA, added to the beads in a final concentration of 50 nM, and incubated for 1 h at room temperature. After addition of the beads to the binding assay, light exposure was omitted and the incubation was carried out for an additional 60 min before analyzing the interaction in the EnVision plate reader (Perkin Elmer).

### Biomolecular modeling.

In order to investigate the structural influences caused by the mutations on the LEDGF/p75 HIV-1 IN interface, a biomolecular modeling approach was used. Structural data of the binding of these two proteins are limited to the crystal structure 2B4J of the HIV-CCD dimer with two IBD fragments [28]. Unresolved loops in the crystal structure were first completed using the B chain of the CCD 1BIS structure [47]. Four different complexes were generated: the WT, the single mutated, and the double mutant structure. Both CCD IN chains of the dimer were mutated. After mutation of the residues and refinement of the mutated residue, a sphere with a radius of 9 Å was subsequently minimized using a CHARMM-based force field [48]. Crystallographic water molecules were incorporated, and an additional water box was used during the energy minimization. In order to analyze the influence of the mutations, the nonbonded interaction energy of the interface and the contribution of the electrostatic energy, Van Der Waals energy, and hydrogen bonding energy were computed for the LEDGF/p75 IN interface and the residues of the interface. Crystallographic water molecules bound in the interface were taken in account for these computations. The computations were performed using MOE (The Molecular Operating Environment, Chemical Computing Group, 1255 University Street, Suite 1600; Montreal, Quebec, Canada H3B 3X3) (modeling) and Brugel [49] (analysis).

## Supporting Information

Figure S1 Effect of eGFP-Δ325 Overexpression on HIV-1 Replication (A) Schematic representation of HIV-1 integration in MT-4 and MT-4 eGFP-Δ325 cells. In MT-4 cells, association with LEDGF/p75 tethers IN to the chromatin, facilitating the integration of the viral cDNA in the host genome. When eGFP-Δ325 is overexpressed, eGFP-Δ325 competes with endogenous LEDGF/p75 for binding to IN and thereby inhibits viral integration. Note that the concentrations of LEDGF/p75 and eGFP-Δ325 proteins shown are arbitrary.
(B) HIV-1 (NL4.3) replication in MT-4 eGFP-Δ325 D366A cells (filled diamonds) and MT-4 eGFP-Δ325 cells (open diamonds). MT-4 cells were infected at an MOI of 0.1, and viral replication was followed by measurement of p24 antigen in the supernatant.
(4.1 MB TIF)

Figure S2 Effect of Resistance Mutations on Viral DNA Formation during Replication (A–C) 293T cells were infected with VSV-G pseudotyped viral clones: WT NL4.3 (diamonds), NL4.3 A128T (boxes), NL4.3 E170G (triangles), or NL4.3 A128T/E170G (crosses). DNA was extracted at different time points postinfection, and the amounts of (A) total viral DNA, (B) 2-LTR circles, and (C) proviruses were determined by quantitative PCR.
(D–F) 293T eGFP-Δ325 and 293T eGFP-Δ325 D366A cells were infected with VSV-G pseudotyped viral clones: WT NL4.3 (diamonds) or NL4.3 A128T/E170G (crosses). DNA was extracted at different time points postinfection, and the amounts of (D) total viral DNA and (E) 2-LTR circles were determined by quantitative PCR. To quantify integrated DNA (F), the total viral DNA was measured 144 h postinfection (i.e., after six cell divisions). Quantifications were performed in duplicate. Averages ± SD are shown.
(4.1 MB TIF)

Figure S3 Effect of Resistance Mutations on Nuclear Localization of mRFP-IN^s^
HeLaP4, HeLaP4 eGFP-Δ325, and HeLaP4 eGFP-Δ325 D366A were transfected with different mRFP-INs^s^, 24 h before laser scanning microscopy.
(A) mRFP-IN^s^ expression in HeLaP4 cells.
(B) mRFP-IN^s^ expression in HeLaP4 eGFP-Δ325 and HeLa P4 eGFP-Δ325 D366A cells.
(C) mRFP-IN^s^ expression upon overexpression of eGFP-LEDGF/p75 in HeLaP4 cells.
(6.4 MB TIF)

Figure S4 Visualization of the IN-IBD Binding Site and Influence of the IN A128T and E170G Mutations Using Biomolecular Modeling Models were drawn with PYMOL (W. L. DeLano, http://www.pymol.org). IN chains A and B are colored blue and green, respectively, while the IBD subunits are violet. Selected residues are shown as sticks, and hydrogen bonds are indicated by dotted lines.
(A) Binding of the two WT catalytic core domains with the IBD (PDB code 2B4J). A128 is part of the α3 helix in the IN-CCD, which forms a hydrophobic patch that accommodates the side chains of the LEDGF residues I365, F406, and V408 [28]. E170 is part of the so-called α4/5 connector, a six-residue connector linking helices α4 and α5 of the second IN chain (residues 166 through 171).
(B) Representation of the model showing the effect of mutating A128 to T128 and E170 to G170. For A128T, the bulky side chain of threonine causes sterical hindrance, thereby forcing the I368 residue of the IBD to adapt a different conformation. This occurs without a drastic influence on the conformation of residues in the LEDGF/p75 IN binding interface. The E170G mutation has a more drastic effect. This mutation results in a different conformation of the loop disrupting two interactions. First, the double hydrogen bond between the hydrogens of the loop backbone at residues E170 and H171 and D366, a critical interacting residue on LEDGF/p75, is broken. Second, the electrostatic interaction of E170 (IN) with K364 (LEDGF/p75) is abolished.
(6.5 MB TIF)

Figure S5 Reduced Replication Kinetics of NL4.3 A128T/E170G Are due to Reduced Affinity for LEDGF/p75 (A) Western blot analysis of LEDGF/p75 expression levels in MT-4 cells (lane 1) and MT-4 cells stably overexpressing LEDGF/p75 (lane 2). As a control, MT-4 cells expressing eGFP were made (MT-4 eGFP) (lane 3). Equal loading was controlled by α-tubulin detection.
(B) MT-4 LEDGF/p75 cells and MT-4 eGFP cells were infected with 10 pg/ml p24 antigen: WT NL4.3 (diamonds) or NL4.3 A128T/E170G (crosses). The replication kinetics for the viruses was determined by measuring viral p24 antigen levels in the supernatant. Experiments were performed in duplicate. Averages ± SD are shown.
(1.3 MB TIF)

### Accession Numbers

The Genbank (http://www.ncbi.nlm.nih.gov/Genbank) accession numbers for the proteins discussed in this paper are LEDGF/p75 (NM_033222) and HIV-1 NL4–3 IN (U26942; base pairs 3608 through 4471).

## Abbreviations

BC: backcomplemented
CCD: catalytic core domain
eGFP: enhanced green fluorescent protein
FCCS: fluorescence cross-correlation spectroscopy
GFP: green fluorescent protein
IBD: integrase binding domain
IN: integrase
MOI: multiplicity of infection
PBL: peripheral blood lymphocyte
PIC: preintegration complex
RFP: red fluorescent protein
VSV-G: vesicular stomatitis virus G protein
WT: wild-type
