# Supplementary figures and images for: Virus Evolution Reveals an Exclusive Role for LEDGF/p75 in Chromosomal Tethering of HIV
Source: PLoS Pathog. 2007 Mar 30;3(3):e47. doi: 10.1371/journal.ppat.0030047 (PMC1839165; doi:10.1371/journal.ppat.0030047)

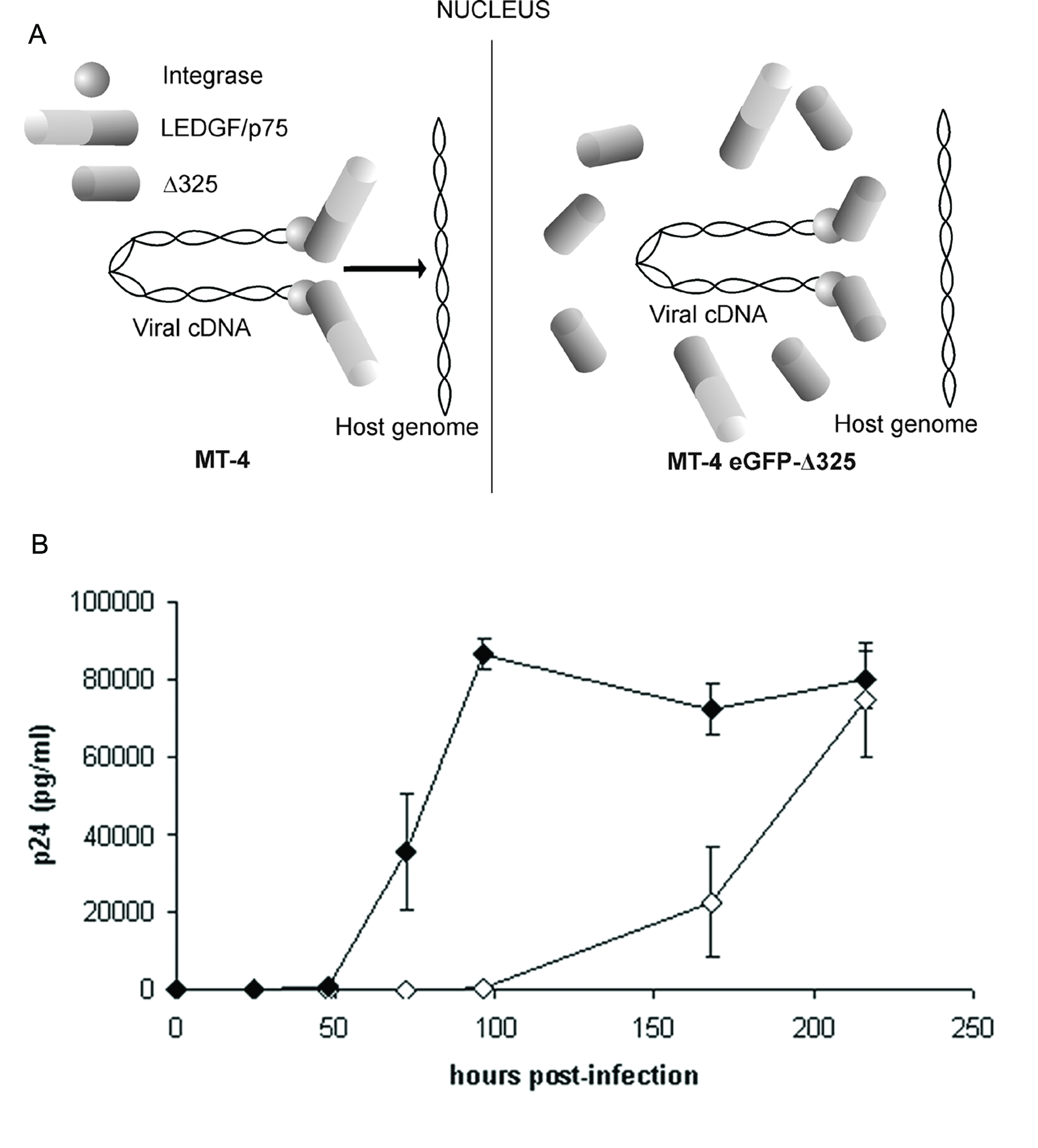

Supplement: Figure S1 — (A) Schematic representation of HIV-1 integration in MT-4 and MT-4 eGFP-Δ325 cells. In MT-4 cells, association with LEDGF/p75 tethers IN to the chromatin, facilitating the integration of the viral cDNA in the host genome. When eGFP-Δ325 is overexpressed, eGFP-Δ325 competes with endogenous LEDGF/p75 for binding to IN and thereby inhibits viral integration. Note that the concentrations of LEDGF/p75 and eGFP-Δ325 proteins shown are arbitrary. (B) HIV-1 (NL4.3) replication in MT-4 eGFP-Δ325 D366A cells (filled diamonds) and MT-4 eGFP-Δ325 cells (open diamonds). MT-4 cells were infected at an MOI of 0.1, and viral replication was followed by measurement of p24 antigen in the supernatant. (4.1 MB TIF) [file ppat.0030047.sg001.tif]

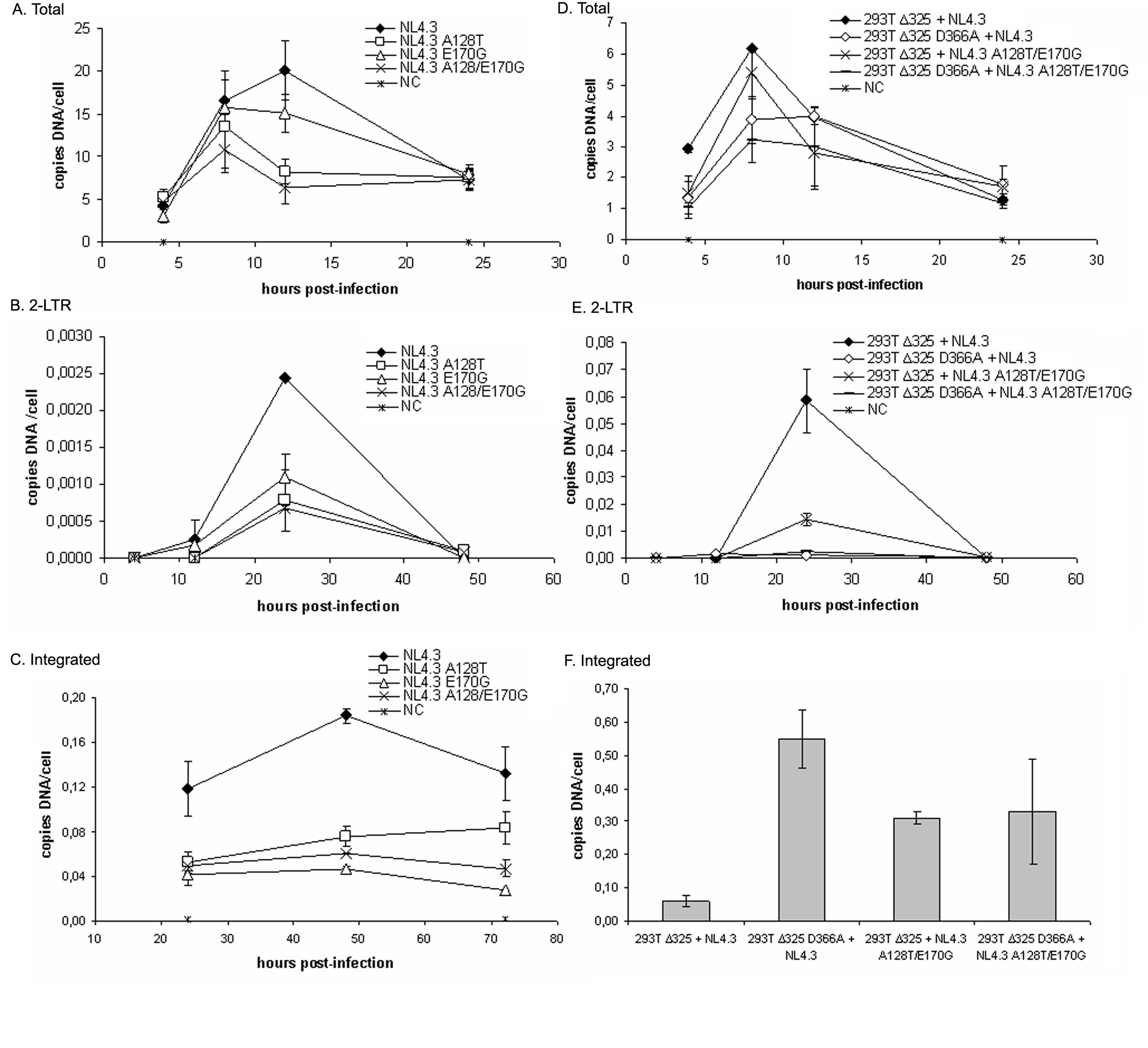

Supplement: Figure S2 — (A–C) 293T cells were infected with VSV-G pseudotyped viral clones: WT NL4.3 (diamonds), NL4.3 A128T (boxes), NL4.3 E170G (triangles), or NL4.3 A128T/E170G (crosses). DNA was extracted at different time points postinfection, and the amounts of (A) total viral DNA, (B) 2-LTR circles, and (C) proviruses were determined by quantitative PCR. (D–F) 293T eGFP-Δ325 and 293T eGFP-Δ325 D366A cells were infected with VSV-G pseudotyped viral clones: WT NL4.3 (diamonds) or NL4.3 A128T/E170G (crosses). DNA was extracted at different time points postinfection, and the amounts of (D) total viral DNA and (E) 2-LTR circles were determined by quantitative PCR. To quantify integrated DNA (F), the total viral DNA was measured 144 h postinfection (i.e., after six cell divisions). Quantifications were performed in duplicate. Averages ± SD are shown. (4.1 MB TIF) [file ppat.0030047.sg002.tif]

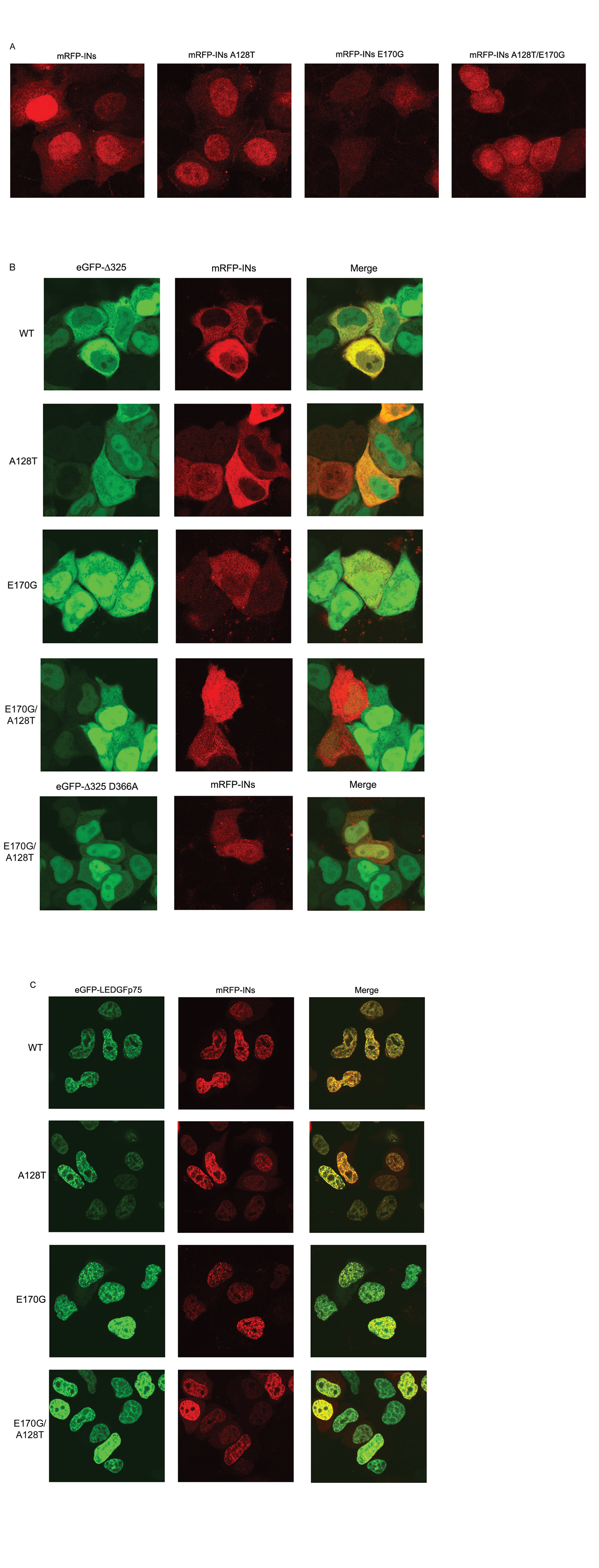

Supplement: Figure S3 — HeLaP4, HeLaP4 eGFP-Δ325, and HeLaP4 eGFP-Δ325 D366A were transfected with different mRFP-INss, 24 h before laser scanning microscopy. (A) mRFP-INs expression in HeLaP4 cells. (B) mRFP-INs expression in HeLaP4 eGFP-Δ325 and HeLa P4 eGFP-Δ325 D366A cells. (C) mRFP-INs expression upon overexpression of eGFP-LEDGF/p75 in HeLaP4 cells. (6.4 MB TIF) [file ppat.0030047.sg003.tif]

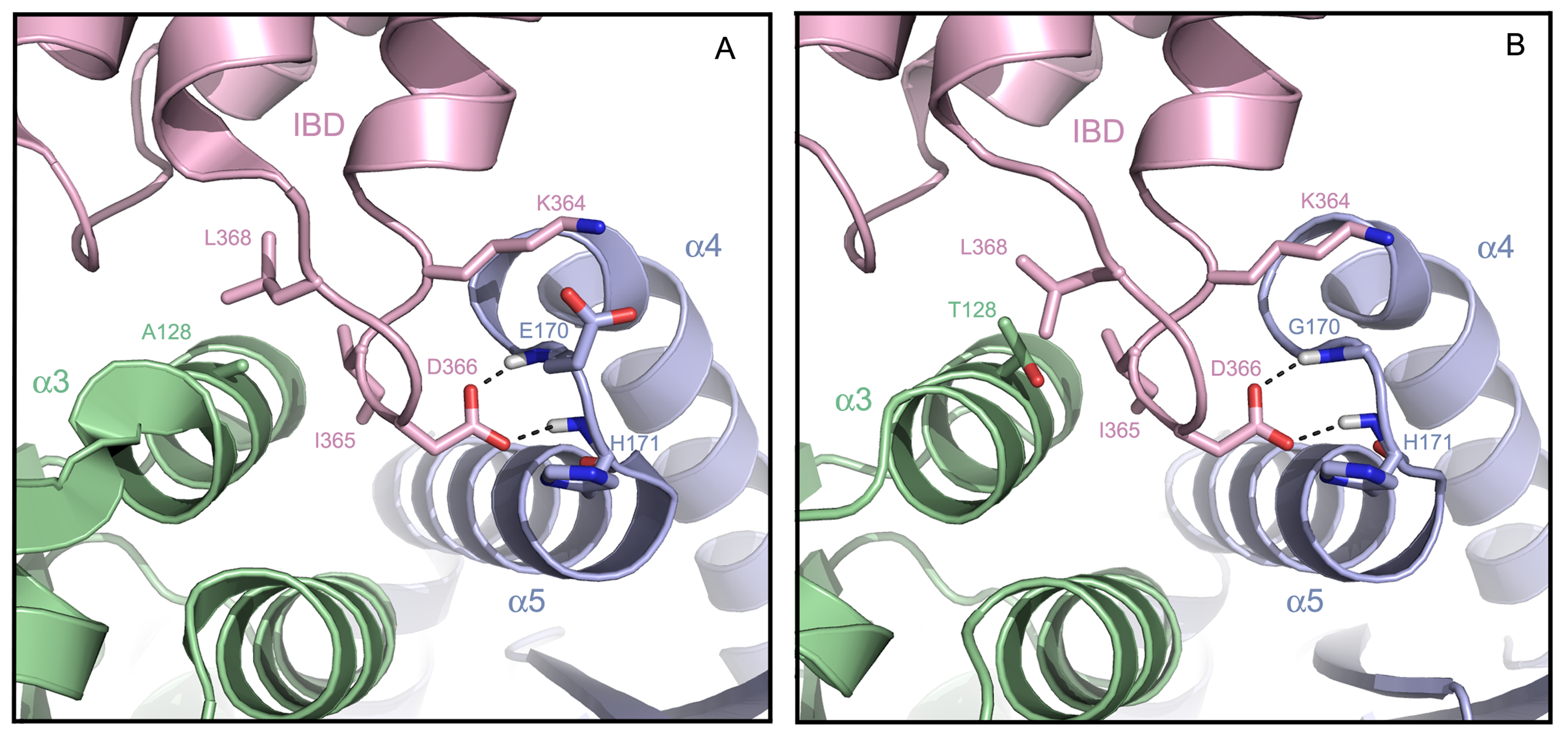

Supplement: Figure S4 — Models were drawn with PYMOL (W. L. DeLano, http://www.pymol.org). IN chains A and B are colored blue and green, respectively, while the IBD subunits are violet. Selected residues are shown as sticks, and hydrogen bonds are indicated by dotted lines. (A) Binding of the two WT catalytic core domains with the IBD (PDB code 2B4J). A128 is part of the α3 helix in the IN-CCD, which forms a hydrophobic patch that accommodates the side chains of the LEDGF residues I365, F406, and V408 [28]. E170 is part of the so-called α4/5 connector, a six-residue connector linking helices α4 and α5 of the second IN chain (residues 166 through 171). (B) Representation of the model showing the effect of mutating A128 to T128 and E170 to G170. For A128T, the bulky side chain of threonine causes sterical hindrance, thereby forcing the I368 residue of the IBD to adapt a different conformation. This occurs without a drastic influence on the conformation of residues in the LEDGF/p75 IN binding interface. The E170G mutation has a more drastic effect. This mutation results in a different conformation of the loop disrupting two interactions. First, the double hydrogen bond between the hydrogens of the loop backbone at residues E170 and H171 and D366, a critical interacting residue on LEDGF/p75, is broken. Second, the electrostatic interaction of E170 (IN) with K364 (LEDGF/p75) is abolished. (6.5 MB TIF) [file ppat.0030047.sg004.tif]

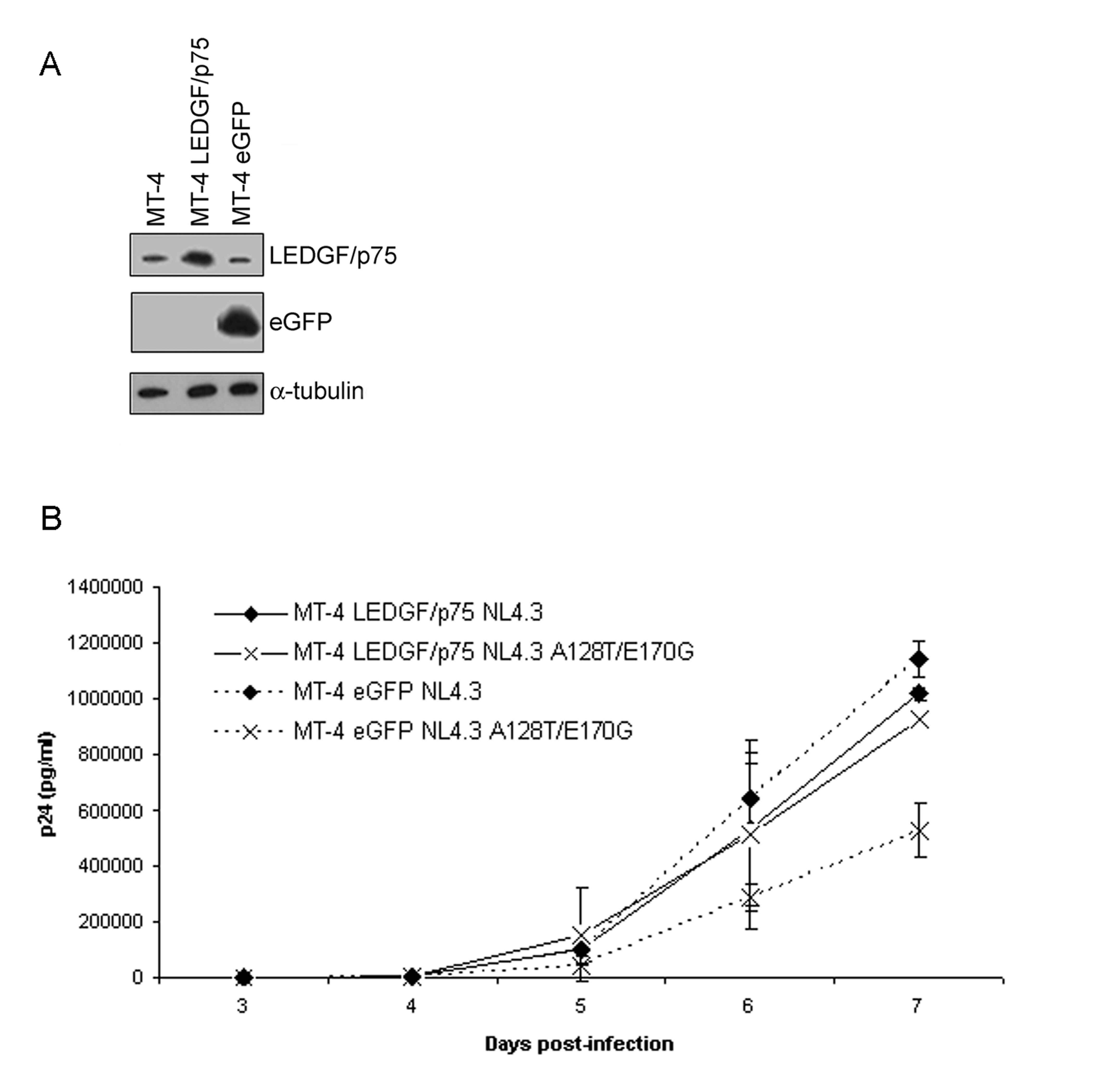

Supplement: Figure S5 — (A) Western blot analysis of LEDGF/p75 expression levels in MT-4 cells (lane 1) and MT-4 cells stably overexpressing LEDGF/p75 (lane 2). As a control, MT-4 cells expressing eGFP were made (MT-4 eGFP) (lane 3). Equal loading was controlled by α-tubulin detection. (B) MT-4 LEDGF/p75 cells and MT-4 eGFP cells were infected with 10 pg/ml p24 antigen: WT NL4.3 (diamonds) or NL4.3 A128T/E170G (crosses). The replication kinetics for the viruses was determined by measuring viral p24 antigen levels in the supernatant. Experiments were performed in duplicate. Averages ± SD are shown. (1.3 MB TIF) [file ppat.0030047.sg005.tif]
